# Refractive Error among Children Visiting the Department of Paediatric Opthalmology of a Tertiary Eye Care Center

**DOI:** 10.31729/jnma.8322

**Published:** 2023-11-30

**Authors:** Govind Gurung, Krishna Kant Gupta

**Affiliations:** 1 Department of Paediatric Opthalmology, RamKumar Mahavir Prasad Kedia Eye Hospital, Parwanipur, Birgunj, Nepal; 2 Department of Glaucoma, RamKumar Mahavir Prasad Kedia Eye Hospital, Parwanipur, Birgunj, Nepal

**Keywords:** *astigmatism*, *hyperopia*, *myopia*, *refractive error*, *visual impairment*

## Abstract

**Introduction:**

Refractive error is the inability of eyes to focus clearly on images. Visual impairment due to refractive error has a major impact on children's education and daily activities. The hospital has no documentation of the ocular morbidity related to refractive errors in children. The aim of this study was to find out the prevalence of refractive error in children visiting the Department of Paediatric Ophthalmology of a tertiary care centre.

**Methods:**

A descriptive cross-sectional study was done in the Outpatient Department of Paediatric Ophthalmology in a tertiary care centre from 8 September 2022 to 7 March 2023 after obtaining ethical approval from the Institutional Review Committee. A convenience sampling method was used. The point estimate was calculated at 95% Confidence Interval.

**Results:**

Among 3600 children, the prevalence of refractive error was seen in 668 children (18.56%) (15.61-21.51, 95% Confidence Interval). Refractive error was seen in 363 (54.34%) boys and 305 (45.66%) girls. Myopia was found in 340 (50.90%), astigmatism in 207 (30.99%), and hyperopia in 121 (18.11%).

**Conclusions:**

The prevalence of refractive error among children attending a tertiary care centre was found to be higher than studies done in similar settings. Regular screening of refractive error for visual impairment is recommended among school going children.

## INTRODUCTION

Refractive errors are the most common cause of vision problems. It is the inability of the eye to focus images clearly from the outside world that results in blurring of vision and visual impairment. Visual impairment ranges in severity from mild visual loss to total absence of light perception.^1,2^ Visual impairment is defined as a functional limitation of the eye(s) or the visual perception.^2^

Uncorrected refractive error is the leading cause of visual impairment in children and is a priority of eye health programs in developing countries.^3,4^ Refractive error study in children (RESC) group mentioned refractive error as the major cause of worsening visual acuity in 56% of children in Nepal.^5^

The aim of the study was to find out the prevalence of refractive error among children attending the visiting the Department of Paediatric Ophthalmology of a tertiary care centre.

## METHODS

This descriptive cross-sectional study was conducted in the Outpatient Department of Paediatric Ophthalmology of RamKumar Mahavir Prasad Kedia Eye Hospital, Parwanipur, Birgunj, Nepal from 8 September 2022 to 7 March 2023 after obtaining ethical approval from the Institutional Review Committee (Reference number: 69-079-080). All the children above 6 years old and below 15 years old attending the outpatient department in the hospital giving consent were included in the study. Children with ocular injury and with ocular pathology were excluded. Convenience sampling method was used. The sample size was calculated using the following formula:


$$\begin{array}{ll} \text{n} & ={\text{Z}}^{2}\times \frac{\text{p}\times \text{q}}{{\text{e}}^{\text{2}}}\\ & =1.96^{2}\times \frac{0.50\times 0.50}{0.05^{2}}\\ & =384 \end{array}$$

Where,

n = minimum required sample size
Z = 1.96 at 95% Confidence Interval (CI)
p = prevalence taken as 50% for a maximum sample calculation
q = 1-p
e = margin of error, 5%

The calculated sample size was 384. However, we have included 3600 patients.

Weighing of children, routine ocular examination including extraocular motility assessment, visual acuity evaluation, cycloplegic refraction, and cover test were done. Uncorrected and best-correction visual acuity (BCVA) was done in an internally illuminated Snellen vision chart at a 6-meter distance. Anterior and posterior segment assessment was done with slit lamp biomicroscopy and an additional 90 Dioptre handheld lens was used for complete fundus evaluation. Children with dioptre more than +1.00 (Hyperopia), -0.5 (Myopia), and ±0.75 (Astigmatism) were included in the study. Demographic details and all other data required for the study were collected from the files of children and the pediatric refractive error register.

Data was entered into Microsoft Excel 2007 and analyzed using IBM SPSS Statistics version 25.0. The point estimate was calculated at a 95% CI.

## RESULTS

Among 3600 children, refractive error was found in 668 (18.56%) (15.61-21.51, 95% CI). A higher frequency of refractive error 393 (58.83%) is seen among the age group 11-15 years (Table 1).

**Table 1:** Age distribution of refractive error (n= 668).

Age group (years)	n (%)
6-10	275 (41.17)
11-15	393 (58.83)

**Figure 1:**
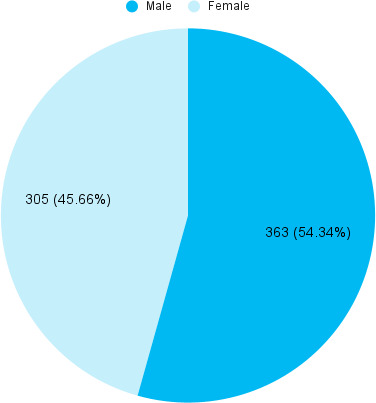
Gender-wise distribution (n= 668).

Among the children with refractive error, 363 (54.34%) were male (Figure 1).

Myopia was found in 340 (50.90%), astigmatism in 207 (30.99%), and hyperopia in 121 (18.11%) making myopia to be the major visual impairment resulting in refractive error (Table 2).

**Table 2:** Visual impairment resulting in refractive error (n= 668).

Visual impairment	n (%)
Myopia	340 (50.90)
Astigmatism	207 (31.11)
Hyperopia	121 (18.11)

Among males, myopia was found in 176 (26.35%) and astigmatism in 102 (15.27%) (Table 3).

**Table 3:** Gender-wise distribution of different types of refractive error (n= 668).

Gender	Myopia n (%)	Astigmatism n (%)	Hyperopia n (%)
Male	176 (26.35)	102 (15.27)	85 (12.72)
Female	164 (24.55)	105 (15.72)	36 (5.39)

## DISCUSSION

The prevalence of refractive error among outpatient children was 18.5%. The prevalence of Myopia was higher than in studies done at Jhapa and Dadeldhura.^5,6^ In a previous study of 3054 children, Myopia was seen in 204 (6.68%) children, hypermetropia in 16 (0.52%) children, and astigmatism in 148 (4.85%) children which is almost similar to our study in which Myopia was seen in 9.4% children followed by astigmatism 5.75%, and hyperopia 3.4%.^7^

The major population of the Parsa and Bara district of Nepal is Madhesi people which comprises various cultural groups such as Hindu caste groups, Muslim, Marwaris, Brahmin and Dalit caste groups, ethnic groups like Maithili, Bhojpuri, Awadi, and Bajjika speaking people. It can explain the differences in results of both refractive error prevalence and myopia prevalence as compared to Jhapa which has a mixed group (Mongol, Indo-Aryan) and Dadeldhura (mostly Mongol). A higher preference for near activities and, an inheritance factor with a decrease in outdoor activities might be the other reasons for an increase in the prevalence of myopia but still major reason remains unknown.^8^ The prevalence of hyperopia (3.36%) and astigmatism (5.75%) was similar to a study conducted in a city in India.^7^ Similarly, it is higher than the previous study done in Pokhara,^9^ and in a multistate study in India which might be due to the smaller population size in this study.^10^

The prevalence of refractive error in this study is 18.5%, which is higher than the study done in Palpa (9%).^11^ Compared to a study done in 15000 children in 2010, myopia was predominant among males and hyperopia was predominant among females, however, this study of 3600 students Myopia was predominant among both males and females followed by astigmatism and hyperopia.^12^ Similarly in a study conducted in 2015 in the Terai region astigmatism was higher among children.^13^ The changes in prevalence in different studies show changing patterns of refractive error. The prevalence is similar to the study done on 4500 children in 2008 in Kathmandu where refractive error was seen in 18.6 percent of children.^14^ According to a previous study myopia progression varies with age and severity of myopia concluded that monitoring of axial length measurement, anti-myopia planning, and consecutive follow-up of children was needed for the greater progression in 'severe myopes' across different age groups to control myopia progression irrespective of the age and degree of myopia.^15^

There is no study done on refractive error in this Parsa and Bara terai belt so this study would give guidance for further research in this area. To avoid duplication of data only one hospital pediatric outpatient department was involved in the study though involvement of another hospital would have added benefits to the study.

Convience sampling method was used so there is a sampling bias. A community based study using simple random sampling method is recommened to increase the generalisability in larger population.

## CONCLUSIONS

The prevalence of refractive error among children visiting the Department of Pediatric Ophthalmology of a tertiary care centre was higher than other studies done in similar settings. Refractive error being a treatable cause of visual impairment, regular screening of children is recommended.
